# Pterostilbene in Cardiovascular Diseases: From Molecular Mechanisms to Therapeutic Potential

**DOI:** 10.3390/biomedicines14040858

**Published:** 2026-04-09

**Authors:** Xin-Fang Leong

**Affiliations:** Department of Craniofacial Diagnostics and Biosciences, Faculty of Dentistry, Universiti Kebangsaan Malaysia, Kuala Lumpur 50300, Malaysia; leongxinfang@ukm.edu.my

**Keywords:** cardiovascular disease, cardioprotection, inflammation, oxidative stress, pterostilbene

## Abstract

Cardiovascular disease continues to impose a substantial global health burden and arises from interconnected pathological processes, including oxidative injury, inflammatory signaling, endothelial dysfunction, metabolic imbalance, and progressive cardiac and vascular structural remodeling. Growing interest has therefore emerged in naturally derived compounds capable of influencing multiple disease pathways simultaneously. Pterostilbene, a dimethoxylated stilbene structurally related to resveratrol, has gained attention due to its enhanced lipophilicity and improved bioavailability. Recent experimental studies have investigated the cardiovascular effects of pterostilbene in both cellular systems and animal models. Evidence from in vitro studies indicates that this compound modulates key regulatory networks involved in cellular energy metabolism, redox homeostasis, endothelial signaling, and stress-associated cardiomyocyte injury. These actions involve pathways linked to 5′ adenosine monophosphate-activated protein kinase and sirtuin-1 signaling, nitric oxide regulation, antioxidant responses, and ferroptosis-related mechanisms. Findings from in vivo investigations further demonstrate protective effects across multiple cardiovascular disease models, including pulmonary hypertension, pressure overload-associated cardiac remodeling, ischemic myocardial injury, toxin-induced cardiotoxicity, and metabolic or atherosclerotic vascular dysfunction. Improvements in functional, structural, and biochemical parameters have been reported in these experimental settings. Overall, current preclinical evidence suggests that pterostilbene may act as a multifunctional modulator of key processes involved in cardiovascular pathology. Although clinical evidence remains limited, the convergence of mechanistic and experimental findings highlights its potential as a multi-target cardiometabolic therapeutic candidate and provides a foundation for future translational and clinical investigation.

## 1. Introduction

Cardiovascular disease (CVD) remains the leading cause of morbidity and mortality worldwide and continues to impose a major clinical and public health burden. Interconnected pathological processes, including oxidative stress, inflammation signaling, endothelial dysfunction, metabolic dysregulation, and maladaptive cardiac and vascular remodeling, drive its development. Despite substantial advances that have been made in the treatment and prevention of CVD, the global burden of CVD continues to rise, underscoring the need for additional therapeutic strategies that can complement current interventions and target multiple disease pathways. Increasing attention has been directed toward naturally derived bioactive compounds, particularly polyphenols, which have demonstrated broad cardiovascular protective effects.

**Figure 1:**
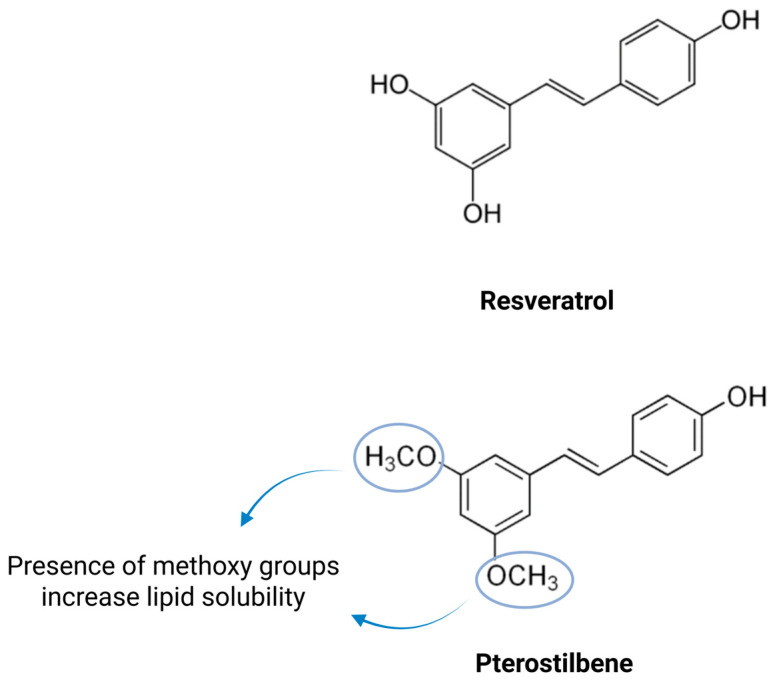
Structural comparison of resveratrol and pterostilbene highlighting the substitution of hydroxyl (–OH) groups with methoxy (–OCH_3_) groups in pterostilbene. Created in BioRender. Leong, X. (2026) https://BioRender.com/78rp3fl, (accessed on 9 March 2026).

Pterostilbene (trans-3,5-dimethoxy-4′-hydroxystilbene) is a naturally occurring stilbenoid identified in several plant sources, including *Pterocarpus marsupium* (heartwood), *Vaccinium* berries, *Vitis vinifera* (grape vine), and *Arachis hypogaea* (peanut) [1,2,3,4]. Structurally related to resveratrol (Figure 1), pterostilbene possesses greater lipophilicity and metabolic stability, which contribute to improved pharmacokinetic properties, including enhanced oral bioavailability and membrane permeability [5,6]. In addition to these favorable characteristics, pterostilbene has been reported to exert a wide range of biological effects, including antioxidant [7,8], anti-inflammatory [9,10], anti-proliferative [11,12], anti-hypertrophic [13,14], and metabolic regulatory effects [15,16]. These properties support its potential relevance in cardiovascular research, where disease progression is shaped by multiple overlapping mechanisms rather than a single dominant pathway.

Over the past decade, an increasing number of experimental studies have investigated the effects of pterostilbene in cellular and animal models. Documented effects have involved cardiomyocytes, endothelial cells, and vascular smooth muscle cells, as well as diverse in vivo models of cardiovascular injury and remodeling, including pulmonary hypertension, atherosclerosis, and heart failure [13,14,17,18,19]. These studies suggest that pterostilbene may confer cardioprotection through multiple mechanisms linked to cellular metabolism, oxidative stress regulation, inflammatory signaling, endothelial homeostasis, and stress-induced cell injury. These findings suggest that pterostilbene may influence several biological processes relevant to cardiovascular dysfunction and remodeling.

Although pterostilbene has been discussed in broader pharmacological reviews and in reviews focused on selected disease contexts [20,21,22,23], a dedicated synthesis integrating molecular mechanisms with functional cardiovascular outcomes across diverse experimental models remains limited. Heterogeneity in study design, disease models, dosing strategies, and experimental endpoints complicates the interpretation of the overall evidence base and its translational significance. Therefore, this narrative review aims to critically examine the available evidence on the cardioprotective actions of pterostilbene, with emphasis on its molecular mechanisms, and therapeutic effects across in vitro and in vivo models, while also considering its translational potential and current limitations.

## 2. Literature Search and Study Selection

A literature search was performed using the Scopus and PubMed databases to identify studies related to pterostilbene and cardiovascular disease. The search strategy used the keywords “pterostilbene” and “cardiovascular”. The search was limited to English-language articles, with no restriction on the year of publication. After the removal of duplicate records, titles and abstracts were screened for relevance, and the full texts of the remaining records were retrieved for further assessment. Original research articles reporting in vitro or in vivo animal studies relevant to cardiovascular mechanisms, injury, or disease models were included. Review articles, editorials, conference abstracts, and studies not directly relevant to pterostilbene or cardiovascular outcomes were excluded. The final included studies were compiled and organized using Microsoft Excel to facilitate structured data extraction and systematic presentation of the findings.

## 3. Molecular Mechanisms in Cardiovascular Pathogenesis

The cardioprotective profile of pterostilbene is underpinned by its capacity to modulate multiple interconnected molecular pathways central to cardiovascular pathogenesis. Mechanistic insights derived from in vitro models, including cardiomyocytes, endothelial cells, and vascular smooth muscle cells, demonstrate that this natural stilbene influences metabolic signaling, redox balance, endothelial homeostasis, and cell survival pathways (Table 1). Collectively, these findings provide a cellular framework for understanding how pterostilbene may mitigate cardiovascular injury. The following subsections delineate the principal molecular mechanisms identified across experimental systems.

### 3.1. AMPK–SIRT1 Signaling and Metabolic Adaptation

In vitro evidence indicates that pterostilbene modulates cellular energy homeostasis primarily through activation of the 5′ adenosine monophosphate-activated protein kinase (AMPK)–sirtuin-1 (SIRT1) signaling axis. In cardiomyocyte models exposed to hypertrophic stimulation, pterostilbene enhances AMPK phosphorylation, thereby promoting adaptive metabolic responses and restoring energy balance [18]. Activation of AMPK is associated with downstream modulation of anabolic and catabolic processes, thereby improving mitochondrial efficiency and attenuating pathological growth signaling [28]. Concurrently, upregulation of SIRT1 has been reported in cardiomyocytes subjected to hypoxia–reoxygenation injury, where pterostilbene enhanced cellular stress resilience by modulating metabolic and survival signaling [26]. Together, these findings suggest that pterostilbene may help preserve cardiomyocyte metabolic homeostasis under stress conditions through activation of energy-sensing pathways relevant to cardiovascular injury.

While metabolic adaptation is critical for maintaining cellular energy balance, persistent cardiovascular stress is also characterized by excessive oxidative injury, which represents another major therapeutic target of pterostilbene.

### 3.2. Redox Regulation and Antioxidant Defense Mechanisms

Beyond metabolic signaling, pterostilbene exerts pronounced effects on cellular redox balance. In cardiomyocyte and isolated cardiac tissue models exposed to oxidative stress, pterostilbene significantly reduces intracellular reactive oxygen species (ROS) accumulation and limits lipid peroxidation [25]. Similarly, in vascular smooth muscle cells, pterostilbene has been shown to reduce hydrogen peroxide (H_2_O_2_) production and counteract oxidized low-density lipoprotein-mediated inhibition of catalase (CAT) activity [19]. These findings indicate that pterostilbene reduces oxidative burden while enhancing endogenous antioxidant defenses, as reflected by decreased ROS/H_2_O_2_ levels and increased antioxidant enzyme activity or expression. However, the extent to which these effects result from direct radical-scavenging versus indirect regulation of redox-related signaling pathways remains to be clarified.

While redox modulation contributes to cellular protection, regulation of vascular cellular signaling represents another critical dimension of pterostilbene’s cardiovascular actions.

### 3.3. Vascular Signaling and Cellular Remodeling

Pterostilbene modulates key pathways governing vascular cellular function and structural remodeling. In vascular smooth muscle cells, pterostilbene inhibits platelet-derived growth factor-BB-induced proliferation by suppressing DNA synthesis and downregulating cell cycle-associated proteins, including cyclins, retinoblastoma protein, proliferating cell nuclear antigen, and cyclin-dependent kinases [27]. These findings suggest a potential role in limiting pathological vascular remodeling.

In endothelial cells, pterostilbene enhances nitric oxide (NO) production through phosphorylation of endothelial nitric oxide synthase, an effect mediated via activation of the phosphoinositide 3-kinase (PI3K)/protein kinase B (Akt) signaling pathway [17]. Notably, inhibition of PI3K/Akt signaling abolishes this protective response, underscoring the specificity of this mechanism [17]. By influencing both vascular tone and remodeling processes, pterostilbene contributes to the maintenance of vascular homeostasis.

More recently, pterostilbene has been shown to inhibit endothelial-to-mesenchymal transition (EndMT) in human pulmonary arterial endothelial cells exposed to profibrotic stimuli [13]. This effect was associated with downregulation of high-mobility group AT-hook proteins (HMGA) and suppression of EndMT-related transcriptional programs, accompanied by preservation of endothelial phenotype [13]. These findings suggest that modulation of the HMGA-dependent EndMT axis represents an additional mechanism by which pterostilbene may attenuate vascular remodeling processes central to cardiovascular pathogenesis. Collectively, these studies suggest that pterostilbene modulates vascular cellular behavior through multiple signaling nodes, although evidence remains limited to selected experimental systems.

When vascular and metabolic stress remain unresolved, progressive structural injury and cardiomyocyte loss may ensue, processes in which regulated cell death pathways play a pivotal role.

### 3.4. Regulation of Ferroptosis and Stress-Induced Cardiomyocyte Injury

Emerging evidence indicates that pterostilbene mitigates stress-induced cardiomyocyte injury through modulation of ferroptotic and mitochondrial injury-related pathways. In angiotensin II-stimulated cardiomyocytes, pterostilbene reduces lipid peroxidation and restores glutathione peroxidase 4 expression, indicating the suppression of iron-dependent ferroptotic cell death [14]. This protective effect was accompanied by the modulation of glycogen synthase kinase-3 beta (GSK-3β) signaling [14], suggesting the involvement of stress-responsive regulatory pathways.

In a model of doxorubicin-induced cardiotoxicity, pterostilbene preserves mitochondrial membrane potential, reduces ultrastructural abnormalities, and limits ROS accumulation [24]. These findings highlight the stabilization of mitochondrial integrity as a key protective mechanism under chemotoxic stress. Collectively, these data indicate that pterostilbene attenuates ferroptotic and stress-mediated injury processes that contribute to cardiomyocyte loss and adverse cardiac remodeling.

## 4. Evidence Across Experimental Cardiovascular Disease Models

The molecular mechanisms described in Section 3 provide a foundation for understanding how pterostilbene exerts protective effects across experimental models of cardiovascular disease. To evaluate its therapeutic relevance, pterostilbene has been investigated across diverse experimental models that recapitulate key features of cardiovascular disease, including pulmonary hypertension, pressure overload-induced cardiac remodeling, ischemic injury, toxin-induced cardiomyopathy, and metabolic or atherosclerotic vascular dysfunction. Although these models vary in species, induction methods, and duration, they collectively reflect fundamental pathological processes, such as endothelial dysfunction, oxidative stress, inflammation, and maladaptive remodeling. The following subsections synthesize evidence from in vivo studies, emphasizing disease-specific outcomes while integrating mechanistic insights to assess the translational potential of this natural stilbene. A summary of the included in vivo studies is provided in Table 2.

### 4.1. Pulmonary Hypertension

Monocrotaline (MCT)-induced pulmonary hypertension models have been widely used to investigate pulmonary vascular remodeling and secondary right-ventricular (RV) dysfunction. Across these models, pterostilbene improved RV functional parameters and attenuated structural remodeling. Enhancements in systolic performance and reductions in RV hypertrophy were consistently observed [13,33,38]. In addition, improvements were reported in myocardial performance index and cardiac output, together with suppression of elevated RV systolic pressure and pulmonary vascular remodeling [13,33,38].

Redox modulation appears to contribute to these protective effects, although regulation of antioxidant systems was not uniformly directional. In the MCT-induced model, pterostilbene reduced the reduced form of nicotinamide adenine dinucleotide phosphate (NADPH) oxidase activity without significantly altering total ROS levels [38], suggesting selective attenuation of enzymatic oxidative sources. Divergence between enzyme activity and protein expression was also reported, as changes in CAT and superoxide dismutase protein levels did not consistently parallel activity-based findings [38]. This apparent inconsistency may reflect the fact that different oxidative and antioxidant endpoints capture distinct aspects of redox regulation, and that enzyme activity does not necessarily parallel protein expression.

In a separate study, enhancement of glutathione-related redox balance and modulation of calcium-handling proteins, including increased sarcoplasmic/endoplasmic reticulum calcium-ATPase expression, were observed [33], supporting improved myocardial contractile regulation. More recent evidence further indicated inhibition of EndMT and suppression of HMGA-dependent transcriptional signaling in pulmonary vascular tissue [13], suggesting preservation of endothelial phenotype as an additional mechanism contributing to reduced vascular remodeling.

Collectively, these findings indicate that pterostilbene attenuates RV dysfunction and pulmonary vascular remodeling in experimental pulmonary hypertension through coordinated effects on redox regulation, myocardial calcium handling, and endothelial phenotypic stability.

### 4.2. Cardiac Hypertrophy and Heart Failure

Chronic pressure overload and hypertensive heart disease are major drivers of cardiac remodeling and progression to heart failure. Experimental models, including transverse aortic constriction (TAC) and spontaneously hypertensive heart failure (SHHF) rats, have provided insight into the potential cardioprotective actions of pterostilbene under sustained hemodynamic stress.

In TAC-induced cardiac hypertrophy, pterostilbene significantly improved left-ventricular systolic function, as evidenced by increased ejection fraction and reduced ventricular dilation, as well as attenuated structural remodeling [14]. These functional benefits were accompanied by modulation of stress-responsive and redox-related signaling pathways [14], supporting a role in mitigating pressure overload-induced maladaptive remodeling.

In contrast, findings in SHHF models were more nuanced. Pterostilbene did not significantly reduce systemic blood pressure or reverse established cardiac hypertrophy [18,37]. However, improvements in indices of ventricular relaxation were observed [18], suggesting enhancement of diastolic function independent of blood pressure reduction. Additionally, vascular structural parameters, including media-to-lumen ratio and arterial stiffness, were favorably modulated [37], indicating potential benefits for resistance-vessel remodeling.

Collectively, these findings suggest that the cardioprotective effects of pterostilbene in pressure overload and hypertensive heart failure models are supportive but context-dependent. Structural regression of hypertrophy appears more evident in surgically induced overload models. In contrast, improvements in ventricular relaxation and vascular stiffness may occur in hypertensive heart failure models even without significant reductions in blood pressure. These heterogeneous responses highlight the complexity of remodeling processes and the importance of disease stage, endpoint selection, and model characteristics in interpreting therapeutic potential.

### 4.3. Ischemic Myocardial Injury

Ischemic myocardial injury, resulting from either sustained coronary occlusion or transient ischemia followed by reperfusion, represents a major contributor to acute cardiac dysfunction and long-term remodeling. Experimental models employing coronary ligation-induced myocardial infarction and ischemia–reperfusion injury have been used to evaluate the cardioprotective potential of pterostilbene.

In coronary ligation-induced myocardial infarction models, pterostilbene attenuated infarct-related structural and functional deterioration, including reductions in infarct size, ventricular remodeling, and indices of oxidative injury [35]. Additional studies further supported its protective effects in this setting, showing improvement in left-ventricular function and redox balance [30], together with favorable effects on right-ventricular and pulmonary parameters in the context of post-infarction cardiopulmonary stress [31]. These benefits were associated with modulation of redox-sensitive pathways, including the activation of nuclear factor erythroid 2-related factor 2 (Nrf2)-related antioxidant responses and regulation of GSK-3β phosphorylation [35], and inhibition of NADPH oxidase-dependent oxidative stress [30,31], suggesting enhanced resistance to oxidative injury during infarct progression.

In ischemia–reperfusion models involving transient coronary occlusion followed by reperfusion, pterostilbene consistently limited infarct expansion and biochemical indices of myocardial injury [36,39,40]. Mechanistically, these cardioprotective effects have been linked to distinct but related pathways, including NO-cGMP-dependent signaling [40], activation of AMPK [36], and suppression of p38 mitogen-activated protein kinase (MAPK) activation with accompanying attenuation of oxidative and inflammatory stress responses [39]. Together, these findings suggest that pterostilbene mitigates reperfusion-associated myocardial injury through integrated redox, metabolic, and signaling-dependent mechanisms.

Collectively, the available evidence demonstrates that pterostilbene attenuates ischemic myocardial damage across both sustained occlusion and reperfusion contexts through multiple, partly overlapping cardioprotective mechanisms.

### 4.4. Drug- and Toxin-Induced Cardiac Injury

Drug- and toxin-mediated cardiac injury models provide insight into cardioprotective strategies under chemically induced stress. Nicotine exposure and doxorubicin administration are both associated with oxidative injury and functional cardiac impairment, thereby serving as relevant platforms to evaluate the protective effects of pterostilbene.

In a nicotine-associated cardiac injury model, pterostilbene reduced systolic, diastolic, and mean arterial blood pressure and improved hemodynamic parameters indicative of left-ventricular dysfunction [32]. These functional improvements were accompanied by attenuation of lipid peroxidation [32], suggesting mitigation of oxidative injury within the cardiac tissue.

In doxorubicin-induced cardiotoxicity, pterostilbene preserved myocardial ultrastructural integrity, including normalization of mitochondrial cristae density and architecture [24]. This structural preservation was associated with reduced ROS generation and enhancement of endogenous antioxidant defenses [24]. Mechanistically, cardioprotection was linked to the activation of AMPK and subsequent upregulation of peroxisome proliferator-activated receptor gamma coactivator 1-alpha and SIRT1 signaling, which are pathways known to regulate mitochondrial biogenesis and cellular energy homeostasis [24]. These findings suggest that pterostilbene mitigates chemotherapy-associated cardiotoxicity through coordinated regulation of mitochondrial function and redox balance.

Collectively, evidence from chemically induced cardiac injury models indicates that pterostilbene mitigates cardiac dysfunction through modulation of oxidative stress, improvement in hemodynamic parameters, and preservation of mitochondrial structure and function. While the nicotine-induced injury model highlights its capacity to attenuate oxidative damage and improve selected cardiovascular parameters, the doxorubicin-induced cardiotoxicity model underscores its role in maintaining mitochondrial integrity and activating metabolic signaling pathways. These findings support a broad cardioprotective potential under conditions of chemical stress, although the dominant mechanisms may vary according to the nature of the injurious stimulus.

### 4.5. Metabolic and Atherosclerotic Cardiovascular Disease

Metabolic dysregulation and atherosclerotic vascular disease represent major contributors to chronic cardiovascular morbidity. Experimental models employing high-fat diet-induced atherosclerosis and fructose-induced metabolic dysfunction have been used to evaluate the cardiometabolic effects of pterostilbene.

In high-fat-diet-induced atherosclerosis, pterostilbene attenuated vascular lipid deposition and structural remodeling in both rabbit and Apolipoprotein E (ApoE)-deficient mouse models [19,29]. These vascular improvements were accompanied by favorable changes in circulating lipid profiles. In high-fat-diet-induced atherosclerotic rabbits, pterostilbene reduced serum total cholesterol and low-density lipoprotein (LDL) levels [29], while in the ApoE-deficient model, plasma total cholesterol and LDL were reported to be reduced in addition to increased high-density lipoprotein levels [19]. Furthermore, a reduction in oxidative stress [19,29] and suppression of inflammatory and smooth muscle-associated remodeling signals [19] were documented. Together, these findings suggest that pterostilbene may mitigate atherosclerotic progression through combined lipid-lowering, antioxidant, and vasculoprotective effects.

Mechanistically, modulation of the PI3K/AKT/mechanistic target of rapamycin (mTOR)-related pathways emerged as a recurring theme. In a high-fat-diet rabbit model, pterostilbene reduced mRNA expression of PI3K, AKT, and mTOR complex 1, while increasing autophagy-associated markers such as microtubule-associated protein 1 light chain 3 beta (LC3B) [29]. Similarly, in ApoE-deficient mice, pterostilbene suppressed activation of AKT and GSK-3β signaling and enhanced phosphatase and tensin homolog deleted on chromosome 10 (PTEN) expression, consistent with the inhibition of proliferative and inflammatory vascular signaling [19]. These findings suggest that pterostilbene may attenuate atherogenesis through coordinated regulation of metabolic and growth-related pathways.

In a fructose-induced diabetic rat model, pterostilbene improved hemodynamic parameters and reduced cardiac hypertrophy while normalizing glucose levels and oxidative stress indices [34]. Suppression of inflammatory signaling pathways, including Nuclear factor-kappa B and nucleotide-binding domain, leucine-rich repeat family, pyrin domain containing 3 (NLRP3)-associated cascades, was accompanied by the activation of AMPK [34]. Increased transcription of mitochondrial respiratory chain-associated genes further suggests the enhancement of mitochondrial regulatory pathways and energy metabolism under metabolic stress [34]. These findings indicate that pterostilbene may reinforce mitochondrial adaptive signaling rather than merely exerting antioxidant effects in diabetic cardiometabolic dysfunction.

Collectively, evidence from metabolic and atherosclerotic models indicates that pterostilbene improves lipid metabolism, suppresses vascular inflammation, and stabilizes atherosclerotic lesions while simultaneously enhancing mitochondrial and antioxidant defenses in metabolic stress contexts. The convergence of PI3K/AKT/mTOR inhibition, AMPK activation, and redox regulation underscores the potential of pterostilbene as a multifunctional modulator of cardiometabolic disease.

Overall, the available in vivo evidence suggests that the most reproducible effects of pterostilbene across cardiovascular disease models include attenuation of oxidative stress, suppression of inflammatory signaling, and improvement in structural or functional indices of tissue injury. These effects are supported by findings from pulmonary hypertension, ischemic myocardial injury, doxorubicin-induced cardiotoxicity, and atherosclerotic or metabolically driven vascular injury models, where pterostilbene was repeatedly associated with reduced oxidative damage, lower inflammatory mediator expression, and preservation of cardiovascular structure or function. At the same time, certain responses appear to be model-specific, such as modulation of ferroptosis-related pathways in doxorubicin injury, NO-cGMP-dependent protection in ischemia–reperfusion, and inhibition of EndMT-related remodeling in pulmonary vascular disease. Pathways supported by multiple studies include redox regulation, inflammatory signaling, mitochondrial preservation, and metabolic stress responses, whereas findings related to antioxidant enzyme expression, hemodynamic improvement, and hypertrophic signaling are less consistent across experimental settings. Taken together, these findings suggest that pterostilbene exerts cardioprotective effects through coordinated modulation of these interconnected pathways, alongside preservation of endothelial integrity, rather than through a single isolated mechanism.

## 5. Therapeutic Potential and Translational Perspectives

### 5.1. Translational Relevance of Preclinical Findings

The translational relevance of pterostilbene lies in the repeated observation of its protective effects across multiple experimental cardiovascular contexts, together with modulation of shared regulatory pathways involved in oxidative stress, inflammation, metabolism, and structural remodeling. Despite variation in model systems, species, and induction methods, several mechanistic themes recur, including attenuation of oxidative stress, suppression of inflammatory signaling, regulation of PI3K/AKT and AMPK pathways, and preservation of mitochondrial and endothelial integrity. The recurrence of these effects across independent disease settings supports the biological plausibility of pterostilbene as a multi-target modulator of cardiometabolic injury rather than a single-target intervention.

Importantly, the reported effects extend beyond changes in biochemical markers to include improvements in structural and functional cardiovascular parameters, such as attenuation of ventricular remodeling, reduction in infarct size, stabilization of atherosclerotic lesions, and improved hemodynamic indices. The ability of pterostilbene to influence both myocardial and vascular compartments suggests a broader regulatory influence that is relevant to the multifactorial nature of CVD.

However, the translation of these findings into human disease requires caution. Reproducibility across preclinical models does not necessarily predict clinical efficacy, as experimental systems cannot fully capture the complexity, chronicity, comorbidity burden, and treatment variability seen in human CVD. Accordingly, while the breadth of preclinical evidence provides a rational basis for further translational investigation, it should be interpreted as supportive rather than definitive evidence for therapeutic positioning in cardiometabolic disorders.

### 5.2. Pharmacokinetics, Safety, and Dose Translation

Resveratrol and pterostilbene are structurally related stilbenes with overlapping reported biological activities, including antioxidant and anti-inflammatory effects relevant to cardiovascular protection. In some experimental settings, pterostilbene has demonstrated greater inhibitory effects on oxidative stress and inflammatory responses than resveratrol [16,41,42]. These differences may be partly explained by the more favorable pharmacokinetic profile of pterostilbene. Compared with resveratrol, pterostilbene exhibits greater lipophilicity, improved membrane permeability, enhanced metabolic stability, higher oral bioavailability, and a longer half-life [5,43,44]. These advantages are largely related to its dimethoxy substitutions, which reduce susceptibility to rapid phase II conjugation and may improve intestinal absorption and hepatic stability [5,6,45]. Pterostilbene is metabolized predominantly through phase II biotransformation, particularly glucuronidation and sulfation, and circulating glucuronide and sulfate conjugates have been detected at higher levels than the parent compound [5,6]. Although these properties support its translational interest, direct evidence that such pharmacokinetic advantages translate into superior cardiovascular efficacy remains limited.

Interpretation of preclinical findings also requires careful consideration of dose translation. Experimental cardiovascular studies have used a broad range of exposures, from low- to high-micromolar concentrations in vitro and approximately 2.5–100 mg/kg/day in vivo, depending on the disease model, route of administration, and treatment duration. This heterogeneity complicates direct cross-study comparison and makes extrapolation to human use challenging. Moreover, the relationship between experimentally effective concentrations and plasma levels achievable in humans has not yet been clearly established.

Available human supplementation data suggest that pterostilbene is generally well tolerated at doses of up to 250 mg/day for 8 weeks, with no major adverse effects reported on hematological, hepatic, renal, thyroid, lipid or glucose-related biochemical markers in randomized, double-blind, placebo-controlled studies [46,47]. However, current evidence remains insufficient to conclude that pterostilbene is suitable for all populations, as long-term safety, use in individuals with substantial comorbidity or polypharmacy, and safety in special populations remain inadequately characterized. Taken together, the pharmacokinetic profile of pterostilbene may offer translational advantages over other natural stilbenes, but rigorous dose-translation studies, pharmacokinetic–pharmacodynamic correlation analyses, and well-designed human cardiovascular trials are still required before its clinical relevance can be established.

## 6. Limitations and Future Research Directions

### 6.1. Limitations of Current Preclinical Evidence

Despite the breadth of experimental findings, several limitations warrant careful consideration when interpreting the cardioprotective potential of pterostilbene. First, the majority of studies have been conducted in rodent models, with limited validation in large-animal systems that more closely reflect human cardiovascular physiology. Interspecies differences in metabolism, disease progression, and pharmacokinetics may influence both efficacy and safety profiles, thereby limiting direct translation.

Heterogeneity in experimental design also presents challenges for comparative interpretation. Variations in dosing regimens, duration of treatment, route of administration, and disease induction protocols make it difficult to define standardized therapeutic windows or directly compare outcomes across studies. Additionally, many investigations focus on short-term interventions, leaving long-term efficacy and safety underexplored, particularly in chronic cardiovascular settings.

An additional limitation is that pterostilbene was predominantly obtained from commercial suppliers, while its source and method of preparation were often not explicitly specified. As a result, direct comparison between naturally derived and synthetic preparations was not possible, and variability in source, purity, or formulation may have contributed to interstudy heterogeneity.

Although mechanistic signaling pathways have been widely examined, direct functional assessment of mitochondrial and metabolic performance remains limited in several models. In many cases, conclusions regarding bioenergetic improvement are inferred from regulatory signaling changes rather than direct measurements of mitochondrial respiration, adenosine triphosphate (ATP) production, or metabolic influx. Similarly, sex-specific responses and age-related variability have been insufficiently examined across studies.

Another limitation is that, while biochemical and structural improvements are frequently reported, relatively few studies assess clinically relevant endpoints such as long-term survival, arrhythmia burden, or progression to overt heart failure. These gaps underscore the need for integrative experimental designs that incorporate functional, molecular, and longitudinal outcome measures.

As a narrative review, this synthesis does not apply a structured risk-of-bias assessment, which may limit the weighting of the included evidence. Together, these limitations indicate that more standardized, mechanistically integrated, and clinically aligned investigations are needed before firm therapeutic conclusions can be drawn.

### 6.2. Future Research Directions

**Figure 2:**
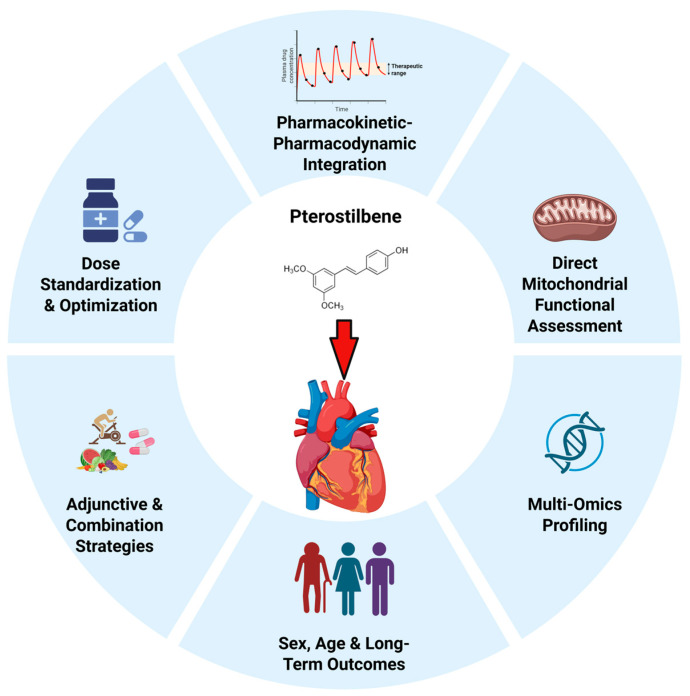
Overview of future research directions for the translational development of pterostilbene in cardiovascular diseases. Created in BioRender. Leong, X. (2026) https://BioRender.com/uvryv87, (accessed on 9 March 2026).

Future investigations should aim to strengthen the translational path from experimental validation to clinical application (Figure 2). Standardization of dosing strategies, treatment duration, formulation, and route of administration will be essential to improve cross-study comparability and facilitate dose-scaling analyses relevant to human use. Integration of pharmacokinetic–pharmacodynamic approaches, including correlation of systemic exposure with mechanistic and functional outcomes, would further help define therapeutic windows and optimize dosing strategies. This is important to determine whether therapeutically relevant exposure levels can be achieved safely and meaningfully in cardiovascular settings.

Mechanistically, greater emphasis should be placed on direct functional assessments of mitochondrial respiration, ATP production, and metabolic flux to complement signaling-based findings. The incorporation of multi-omics approaches, including transcriptomics, metabolomics, and proteomics, may also help clarify the broader regulatory networks influenced by pterostilbene. In addition, future studies should examine sex-specific responses, age-dependent effects, and long-term intervention outcomes to improve relevance to heterogeneous patient populations.

Given the multifactorial nature of CVD, exploration of combination strategies may also be warranted. Evaluating pterostilbene alongside established pharmacotherapies or lifestyle interventions could help determine whether it is more effective as an adjunctive modulator than as a standalone therapeutic agent. Finally, early-phase clinical studies in cardiometabolic risk states, such as metabolic syndrome, early atherosclerosis, or subclinical ventricular dysfunction, may provide practical entry points for translational evaluation. Progress in these areas will be essential for determining whether the preclinical cardioprotective effects of pterostilbene can be translated into clinically meaningful benefit.

## 7. Conclusions

Pterostilbene, a naturally occurring stilbene with favorable pharmacological properties, has emerged as a promising cardiometabolic modulator supported by a growing body of preclinical evidence. Across cellular and animal models, pterostilbene has frequently been reported to attenuate oxidative stress, inflammatory signaling, maladaptive remodeling, and metabolic dysregulation, with corresponding improvements in functional and structural cardiovascular outcomes.

The convergence of redox regulation, AMPK activation, PI3K/AKT pathway modulation, endothelial stabilization, and mitochondrial regulatory signaling suggests that pterostilbene may influence several key pathways in cardiovascular pathogenesis. Although these findings support its mechanistic relevance and translational potential, further standardized preclinical studies and well-designed clinical investigations are required to clarify optimal dosing, long-term safety, and therapeutic applicability. Overall, pterostilbene remains a biologically plausible and mechanistically supported candidate for future cardiometabolic research and intervention.

## Figures and Tables

**Table 1:** Summary of in vitro and ex vivo studies on the protective effects of pterostilbene in cardiovascular cell and tissue models.

Author (Year)	Cell/Tissue Type	Experimental Condition	Concentration of Pterostilbene	Findings	Reported Mechanisms
Wang et al. (2025) [13]	hPAECs	TGF-β/TNF-α/IL-1β-induced EndMT	20 μM	↑ PECAM-1, vWF ↓ α-SMA expression ↓ Collagen 1, fibronectin Restoration of NO production	Inhibition of HMGA1/2 Inhibition of Snai1/2, Twist1
Zhang et al. (2024) [14]	Primary cardiac myocytes, H9C2 cells	Ang-II-induced ferroptosis	100 μg/mL	↓ ROS, lipid peroxidase ↓ Fe^2+^ accumulation ↑ Ferritin expression	Activation of SIRT1/GSK-3β/GPX4
Liu et al. (2019) [24]	H9C2 cells	Doxorubicin-induced injury	10 μM	↑ Cell viability ↓ ROS Preservation of mitochondrial membrane potential and ATP content	Activation of AMPK and upregulation of PGC-1α Activation of SIRT1 with deacetylation of PGC-1α
Wang et al. (2019) [19]	Mouse vascular aortic smooth muscle cells	Oxidized LDL exposure	0.1, 0.5, 1 μM	↓ VSMC proliferation ↓ H_2_O_2_ production ↑ CAT activity and expression	Inhibition of Akt/PRAS40/GSK-3β Upregulation of PTEN
Couto et al. (2018) [25]	H9C2 cells, cardiac tissue	H9C2: H_2_O_2_-induced oxidative stress	50 μM	↑ Cell viability ↑ Sulfhydryl levels ↓ ROS ↑ SOD, CAT ↓ Nitrite	Activation of antioxidant enzymes
		Tissue: Hydroxyl radicals exposure	25, 50 μM		
Akinwumi et al. (2017) [18]	Neonatal ventricular myocytes	Endothelin-1-induced hypertrophy	1 μg/mL	↓ Myocyte enlargement	Activation of AMPK
Guo et al. (2016) [26]	H9C2 cells	Ischemia–reperfusion injury	3 μM	↓ Cell death	Activation of SIRT1
Park et al. (2015) [17]	HUVECs	Basal condition	10 μM	↑ NO production	Activation of PI3K/Akt
Park et al. (2010) [27]	VSMCs	PDGF-BB-induced proliferation	1, 3, 5 μM	↓ VSMCs proliferation ↓ CDK2, Cyclin E, CDK4, Cyclin D1, Rb, PCNA ↓ DNA synthesis	Inhibition of Akt

Abbreviations: α-SMA: alpha-smooth muscle actin; Akt: protein kinase B; AMPK: 5′ adenosine monophosphate-activated protein kinase; Ang II: angiotensin II; ATP: adenosine triphosphate; CAT: catalase; CDK: cyclin-dependent kinase (e.g., CDK2, CDK4); DNA: deoxyribonucleic acid; EndMT: endothelial-to-mesenchymal transition; GPX4: glutathione peroxidase 4; GSK-3β: glycogen synthase kinase-3 beta; H_2_O_2_: hydrogen peroxide; HMGA: high-mobility group A (e.g., HMGA1, HMGA2); hPAECs: human pulmonary artery endothelial cells; IL-1β: interleukin-1 beta; LDL: low-density lipoprotein; NO: nitric oxide; PCNA: proliferating cell nuclear antigen; PDGF-BB: platelet-derived growth factor-BB; PECAM: platelet endothelial cell adhesion molecule; PGC-1α: peroxisome proliferator-activated receptor gamma coactivator 1-alpha; PI3K: phosphoinositide 3-kinase; PRAS40: proline-rich Akt substrate of 40 kDa; PTEN: phosphatase and tensin homolog deleted on chromosome 10; Rb: retinoblastoma protein; ROS: reactive oxygen species; SIRT1: sirtuin 1; Snail: snail family transcriptional repressor (e.g., Snail1, Snail2); SOD: superoxide dismutase; TGF-β: transforming growth factor-beta; TNF-α: tumor necrosis factor-alpha; Twist1: twist family basic helix–loop–helix transcription factor 1; VSMC: vascular smooth muscle cell; vWF: Von Willebrand factor; ↑: increased; ↓ decreased.

**Table 2:** Summary of in vivo studies on the cardiovascular effects of pterostilbene in experimental disease models.

Author (Year)	Animal Model	Disease Induction	Dose, Route of Administration and Duration	Outcomes	Reported Mechanisms
Sahib et al. (2025) [29]	1- to 3-year-old male and female New Zealand white rabbits	High-fat diet (2% cholesterol)-induced atherosclerosis	10 mg/kg/day, oral, 8 weeks	↓ serum TC, LDL ↓ serum F_2_-isoprostane ↓ Aortic lipid accumulation and intimal thickening ↑ Aortic LC3B	PI3K/Akt/mTORC1 inhibition Autophagy activation
Wang et al. (2025) [13]	8- to 10-week-old male Sprague-Dawley rats	Monocrotaline-induced pulmonary hypertension	15 mg/kg/day, oral, 25 days	↓ RVSP and RV hypertrophy ↑ Lumen area	HMGA1/2 inhibition Inhibition of Snai1/2, Twist1
Zhang et al. (2024) [14]	10-week-old male C57BL/6 mice	Transverse aortic constriction-induced heart failure	2.5 mg/kg/day, oral, 8 weeks	↑ LVEF%, LVFS%, CO ↓ LVID ↓ Cardiomyocyte size ↓ Cardiac collagen volume fraction	Activation of SIRT1/GSK-3β/GPX4
de Castro et al. (2024) [30]	Young adult male Wistar rats	Coronary ligation-induced acute myocardial infarction	100 mg/kg/day, oral, 8 days	↑ LVEF, ↓ LV end systolic volume ↓ LV lipid peroxidation ↑ LV GSH	Inhibition of NADPH-oxidase-dependent oxidative stress
		Monocrotaline-induced pulmonary hypertension	100 mg/kg/day, oral, 14 days	↑ pulmonary artery flow ↓ RV hypertrophy ↓ RV ROS ↑ RV SOD, CAT, GSH	
Tasca et al. (2022) [31]	Young adult male Wistar rats	Coronary ligation-induced acute myocardial infarction	100 mg/kg/day, oral, 8 days	↓ RV end systolic volume, pulmonary congestion ↑ RV sulfhydryl content, NOS activity ↓ RV xanthine oxidase expression ↑ Lung GSH, SOD, CAT ↑ Lung Nrf2 expression	Inhibition of NADPH-oxidase-dependent oxidative stress
Ghazali et al. (2021) [32]	Adult male Sprague-Dawley rats	Nicotine-induced cardiac injury	10 mg/kg/day, i.p., 28 days	↓ SBP, DBP, MAP, HR ↑ LVDP ↓ TBARS	Oxidative stress attenuation
Lacerda et al. (2020) [33]	Adult male Wistar rats	Monocrotaline-induced pulmonary hypertension	25, 50, 100 mg/kg/day, oral, 14 days	↓ RVSD, RVDD, MPI ↑ Systolic and cardiac output ↑ RV GSH, GSR, GST, GRx ↓ RV TBARS ↓ total phospholamban expression ↑ SERCA expression	Activation of non-enzymatic antioxidant defense ↑ Calcium reuptake by upregulating SERCA
Wang et al. (2019) [19]	7-week-old male ApoE^−/−^ mice	High-fat diet (1.25% cholesterol)-induced atherosclerosis	30 mg/kg/day, i.g., 16 weeks	↓ Plaque size and lesion in thoracic and abdominal aorta ↓ Body weight, ↓ Plasma TC, LDL ↑ Plasma HDL ↓ Plasma IL-6. IFN-γ, TNF-α ↓ Aortic MDA and H_2_O_2_ ↑ Aortic CAT activity and expression ↓ Aortic α-SMA expression	Inhibition of Akt/ PRAS40/GSK-3β Upregulation of PTEN
Liu et al. (2019) [24]	8-week-old male C57BL/6 mice	Doxorubicin-induced cardiac injury	10 mg/kg/day, i.p., 7 days	Normalized cristae density and architecture ↓ Cardiac ROS ↑ Cardiac SOD and GPx ↑ Cardiac NRF1, UCP2 expression	Activation of AMPK and upregulation of PGC-1α Activation of SIRT1 with deacetylation of PGC-1α
Kosuru et al. (2018) [34]	3-week-old male Sprague-Dawley rats	High-fructose diet (65%)-induced diabetes	20 mg/kg/day, oral, 8 weeks	↓ Body weight, MAP, HR, cardiac hypertrophy ↓ Blood glucose, serum LDH, CK-MB, AST ↓ Cardiac TBARS, ROS, H_2_O_2_, peroxynitrite ↑ Cardiac SOD, CAT, GSH, GPx ↓ Cardiac and plasma IL-1β, IL-6, TNF-α ↓ Cardiac NF-κB, TLR-4, NLRP3, ASC expression	Activation of AMPK/Nrf2/HO-1
Lacerda et al. (2018) [35]	Adult male Wistar rats	Coronary ligation-induced acute myocardial infarction	100 mg/kg/day, oral, 8 days	↓ LV systolic and diastolic diameter, infarct size ↑ Fractional area change ↓ LV lipid peroxidation and TBARS ↑ LV GSH, TrxR ↓ LV GRx	Modulation of thiol-dependent enzymes Activation of Nrf2/GSK-3β
Kosuru et al. (2018) [36]	Adult male Sprague-Dawley rats	Coronary ligation-induced ischemia–reperfusion injury	20, 40 mg/kg/day, oral, 4 weeks before induced ischemia–reperfusion	↓ Infarct size, apoptosis ↓ Plasma LDH, CK-MB, 8-isoprostane ↑ Cardiac AMPK phosphorylation	Activation of AMPK
Akinwumi et al. (2017) [18]	7-week-old male lean spontaneously hypertensive heart failure rats	Genetic model of hypertension-induced heart failure	2.5 mg/kg/day, oral, 8 weeks	↓ LV IVRT No effect on BP and cardiac hypertrophy	No AMPK activation observed
Lee et al. (2017) [37]	7-week-old male lean spontaneously hypertensive heart failure rats	Genetic model of hypertension-induced heart failure	2.5 mg/kg/day, oral, 8 weeks	↓ Media-to-lumen ratio in mesenteric and cerebral arteries ↓ Arterial wall stiffness No effect on BP and vascular compliance	No AMPK or ERK activation observed
Dos Santos Lacerda et al. (2017) [38]	Adult male Wistar rats	Monocrotaline-induced pulmonary hypertension	25, 50, 100 mg/kg/day, oral, 14 days	↑ FAC, TAPSE, RVEDP ↓ RV weight, RV/LV weight, RV/tibia index ↑ RV SOD, GPx ↓ RV SOD, CAT expression No effect on pulmonary and hepatic congestion No effect on ROS and sulfhydryl content	Inhibition of NADPH-oxidase-dependent oxidative stress
Yu et al. (2017) [39]	Adult male Sprague-Dawley rats	Coronary ligation-induced ischemia–reperfusion injury	10 mg/kg, i.v., 10 min before reperfusion	↓ Infarct size ↓ Cardiac superoxide, MDA, MPO, TNF-α, IL-1β ↑ Cardiac SOD ↓ Cardiac inducible NOS ↑ Cardiac p-endothelial NOS expression	Inhibition of p38 MAPK activation
Lv et al. (2015) [40]	Adult male Sprague-Dawley rats	Coronary ligation-induced ischemia–reperfusion injury	100 µmol/L, i.v., 5 min before reperfusion	↓ Infarct size ↓ Cardiac MPO ↓ Serum CK, LDH ↓ Serum and tissue TNF-α	NO–cGMP–dependent cardioprotection

Abbreviations: α-SMA: alpha-smooth muscle actin; Akt: protein kinase B; AMPK: 5′ adenosine monophosphate-activated protein kinase; ASC: apoptosis-associated Speck-like protein containing a caspase recruitment domain; AST: aspartate aminotransferase; BP: blood pressure; CAT: catalase; cGMP: cyclic guanosine monophosphate; CK-MB: creatine kinase-myocardial band; CO: cardiac output; DBP: diastolic blood pressure; ERK: extracellular signal-regulated kinase; FAC: fractional area change; GPX4: glutathione peroxidase 4; GRx: glutaredoxin; GSH: glutathione; GSK-3β: glycogen synthase kinase-3 beta; GSR: glutathione reductase; GST: glutathione-S-transferase; H_2_O_2_: hydrogen peroxide; HDL: high-density lipoprotein; HMGA: high-mobility group A (e.g., HMGA1, HMGA2); HO-1: heme oxygenase-1; HR: heart rate; IFN-γ: interferon-gamma; i.g.: intragastric; IL: interleukin (e.g., IL-1β, IL-6); i.p.: intraperitoneal; i.v.: intravenous; IVRT: isovolumic relaxation time; LC3B: microtubule-associated protein 1 light chain 3 beta; LDH: lactate dehydrogenase; LDL: low-density lipoprotein; LV: left ventricle; LVDP: left-ventricular developed pressure; LVEF: left-ventricular ejection fraction; LVFS: left-ventricular fractional shortening; LVID: left-ventricular internal dimensions; MAP: mean arterial pressure; MAPK: mitogen-activated protein kinase; MDA: malondialdehyde; MPI: myocardial performance index; MPO: myeloperoxidase; mTORC1: mechanistic target of rapamycin complex 1; NADPH: reduced form of nicotinamide adenine dinucleotide phosphate; NF-κB: Nuclear factor-kappa B; NLRP3: nucleotide-binding domain, leucine-rich repeat family, pyrin domain containing 3; NO: nitric oxide; NOS: nitric oxide synthase; NRF1: nuclear respiratory factor 1; Nrf2: nuclear factor erythroid 2-related factor 2; PGC-1α: peroxisome proliferator-activated receptor gamma coactivator 1-alpha; PI3K: phosphoinositide 3-kinase; PRAS40: pro-line-rich Akt substrate of 40 kDa; PTEN: phosphatase and tensin homolog deleted on chromosome 10; ROS: reactive oxygen species; RV: right ventricle; RVDD: right-ventricular diastole diameter; RVEDP: right-ventricular end-diastolic pressure; RVSD: right-ventricular systole diameter; RVSP: right-ventricular systolic pressure; SBP: systolic blood pressure; SERCA: sarcoplasmic/endoplasmic reticulum calcium-ATPase; SIRT1: sirtuin 1; Snail: snail family transcriptional repressor (e.g., Snail1, Snail2); SOD: superoxide dismutase; TAPSE: tricuspid annular plane systolic excursion; TBARS: thiobarbituric acid reactive substances; TC: total cholesterol; TLR-4: Toll-like receptor 4; TNF-α: tumor necrosis factor-alpha; TrxR: thioredoxin reductase; Twist 1: twist family basic helix–loop–helix transcription factor 1; UCP2: uncoupling Protein 2; ↑: increased; ↓: decreased.

## Data Availability

No new data were created or analyzed in this study. Data sharing is not applicable to this article.
